# Supplementary material for: Inferential planning in the frontal cortex
Source: Cell Rep. 2026 Aug 28;45(9):117905. doi: 10.1016/j.celrep.2026.117905 (PMC13597664; doi:10.1016/j.celrep.2026.117905)
Supplement: Supplementary file 1 [file mmc1.pdf]

Cell Reports, Volume *45*

Supplemental information

Inferential planning in the frontal cortex

Francesco Donnarumma, Thomas Parr, Karl Friston, James Whittington, and Giovanni Pezzulo

Simulation	Policies	Number of policies
Simulation (1):<br>Cursor movements<br>(Mushiake et al., 2006)	All policies have length $T^{(2)} = 3$.<br>The start state is always S_{33}.<br>The goal state varies from trial to trial.	16
Simulation (2):<br>Sequence of saccades<br>(Xie et al., 2022)	All policies have length $T^{(2)} = 3$.<br>Targets are $\{A, \ldots, F\}$, without repetition.	$6P3 = 120$
Simulation (3):<br>Variable-length forward/<br>backward sequence of saccades<br>(Chen et al., 2024)	Policies have lengths $1, 2, 3$.<br>Targets are $\{A, \ldots, F\}$, without repetition.<br>Two subsets of policies for *Forward* and *Backward* sequences.<br>Context (forward or backward) known in advance.	$6P1 + 6P2 + 6P3 = 156$ forward<br>$6P1 + 6P2 + 6P3 = 156$ backward<br>Total = 312 policies
Simulation (4):<br>Fixed-length forward/<br>backward sequence of saccades<br>(Chen et al., 2024)	All policies have length $T^{(2)} = 2$.<br>Targets are $\{A, \ldots, F\}$, without repetition.<br>Two subsets of policies for *Forward* and *Backward* sequences.<br>Context inferred from a cue at t_{cue}.	$6P2 = 30$ forward<br>$6P2 = 30$ backward<br>Total = 60 policies

Table S1 Policies used in the four simulations. At Level 2, a policy is defined as a sequence of control states. Simulation (1) addresses the cursor-movement task of (Mushiake et al., 2006), in which monkeys plan a sequence of cursor movements from the central start state S_{33} to a known goal state that changes across problems. Only policies compatible with the maze topology are allowed; this constraint is encoded as a policy prior, $\mathbf{E}^{(2)} = p(\pi \mid \text{Maze})$. In our simulations, the admissible policies are all valid three-step paths that do not revisit the same state: URR, ULL, DRR, DLL, URD, ULD, DRU, DLU, UUR, UUL, DDR, DDL, DRD, DLD, URU, and ULU. For each problem, the goal state is specified as a preferred outcome, and the best policy is inferred using standard active inference methods (Parr et al., 2022). Simulation (2) addresses the sequence-memory task of (Xie et al., 2022), in which monkeys infer and maintain a sequence of three saccades. Policies correspond to ordered sequences of three targets drawn from A–F, without repetition; examples include ABC, ABE, and ABF, yielding $6P3 = 120$ policies. Simulation (3) addresses the variable-length forward/backward saccade task of (Chen et al., 2024). Policies have length 1, 2, or 3, and the full policy space comprises two sets of 156 policies, one for the *Forward* context and one for the *Backward* context. Because the execution context is known at the beginning of the task, the policy prior selects the relevant subset from the outset. The prior also assigns equal total probability mass to policies of lengths 1, 2, and 3, compensating for the larger number of longer sequences. Simulation (4) addresses the fixed-length cue-dependent task of (Chen et al., 2024). Policies have length 2, and the same target pair can be executed either forward or backward depending on the cue observed at t_{cue}, yielding 60 policy–context combinations. Before the cue, the policy prior is asymmetric: *Forward* policies are one order of magnitude more probable than *Backward* policies, $w_{\text{Forw}} = 10\, w_{\text{Back}}$. In the table, $nPk = n!/(n-k)!$ denotes the number of ordered permutations of k elements chosen from n.

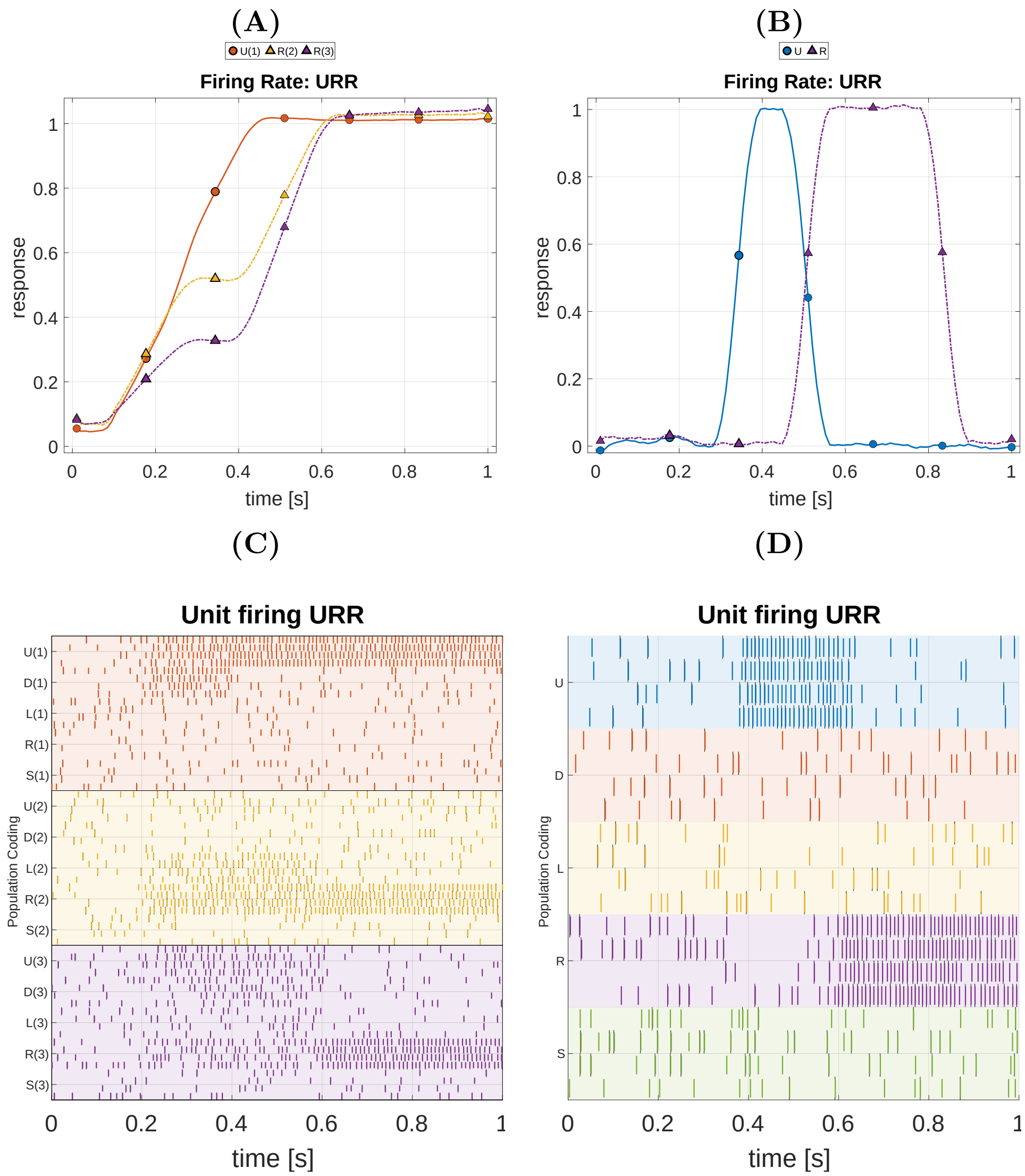

Figure S1 Extended Simulation (1): hidden control states at both levels of the hierarchical generative model during inference of the $U(1), R(2), R(3)$ policy. This figure complements Figure 2G–I of the main text by showing an illustrative simulation in which the policy $U(1), R(2), R(3)$ is inferred from the start location S_{33} to the goal location S_{25}. The panels report neural readouts associated with hidden control states, i.e., actions, during the planning phase. Panels (A) and (B) show firing rates associated with hidden control-state inference at Level 2 and Level 1, respectively. Panels (C) and (D) show the corresponding raster plots at Level 2 and Level 1. The Level 2 inference is identical to that shown in the main text, but here only the firing rates associated with the inferred plan are displayed. The Level 1 inference illustrates the dynamics of hidden states associated with actions to be executed. The figure highlights the distinction between persistent and parallel activation of multiple elements of an action plan at Level 2 and transient, sequential activation of inferred actions at Level 1. In standard active inference simulations, policy inference is interleaved with action execution, typically by executing the first action of the inferred policy, acquiring a new observation, and replanning until the goal is achieved (Parr et al., 2022). That execution phase is not shown here, but it would follow the same transient and sequential dynamics illustrated at Level 1.

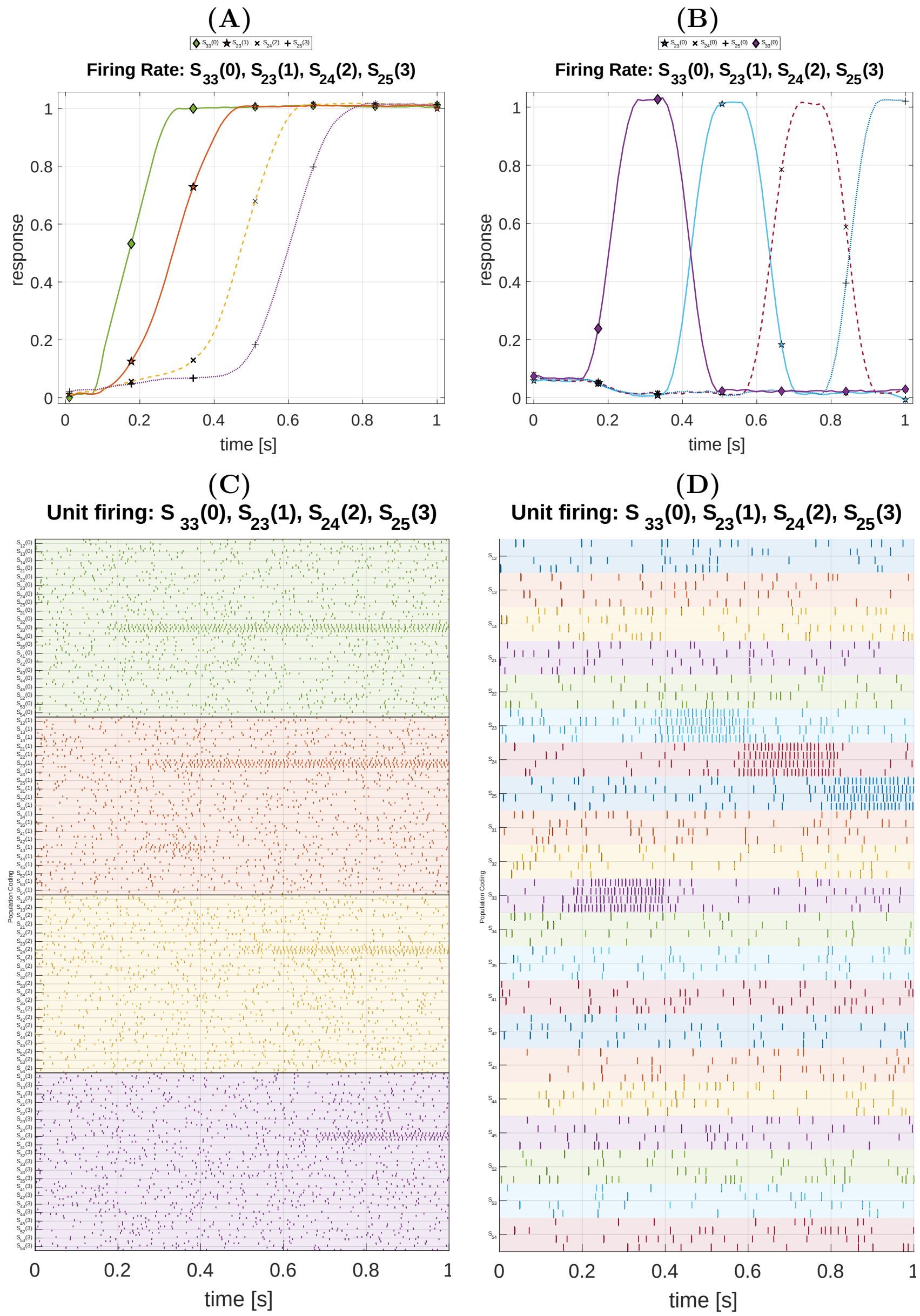

Figure S2 Extended Simulation (1): hidden location states at both levels of the hierarchical generative model during inference of the $U(1), R(2), R(3)$ policy. This figure shows the dynamics of a component of the generative model not shown in the main text: hidden location states during inference of the policy $U(1), R(2), R(3)$. Panels (A) and (B) show firing rates associated with hidden location-state inference at Level 2 and Level 1, respectively. Panels (C) and (D) show the corresponding raster plots at Level 2 and Level 1. Hidden location states correspond to the possible locations of the maze, labeled from S_{12} to S_{54} as in Figure 2G of the main text. The figure highlights the distinction between persistent and parallel activation of multiple locations corresponding to the states expected to be visited while executing the inferred policy at Level 2 and transient, sequential activation of hidden states encoding inferred locations at Level 1.

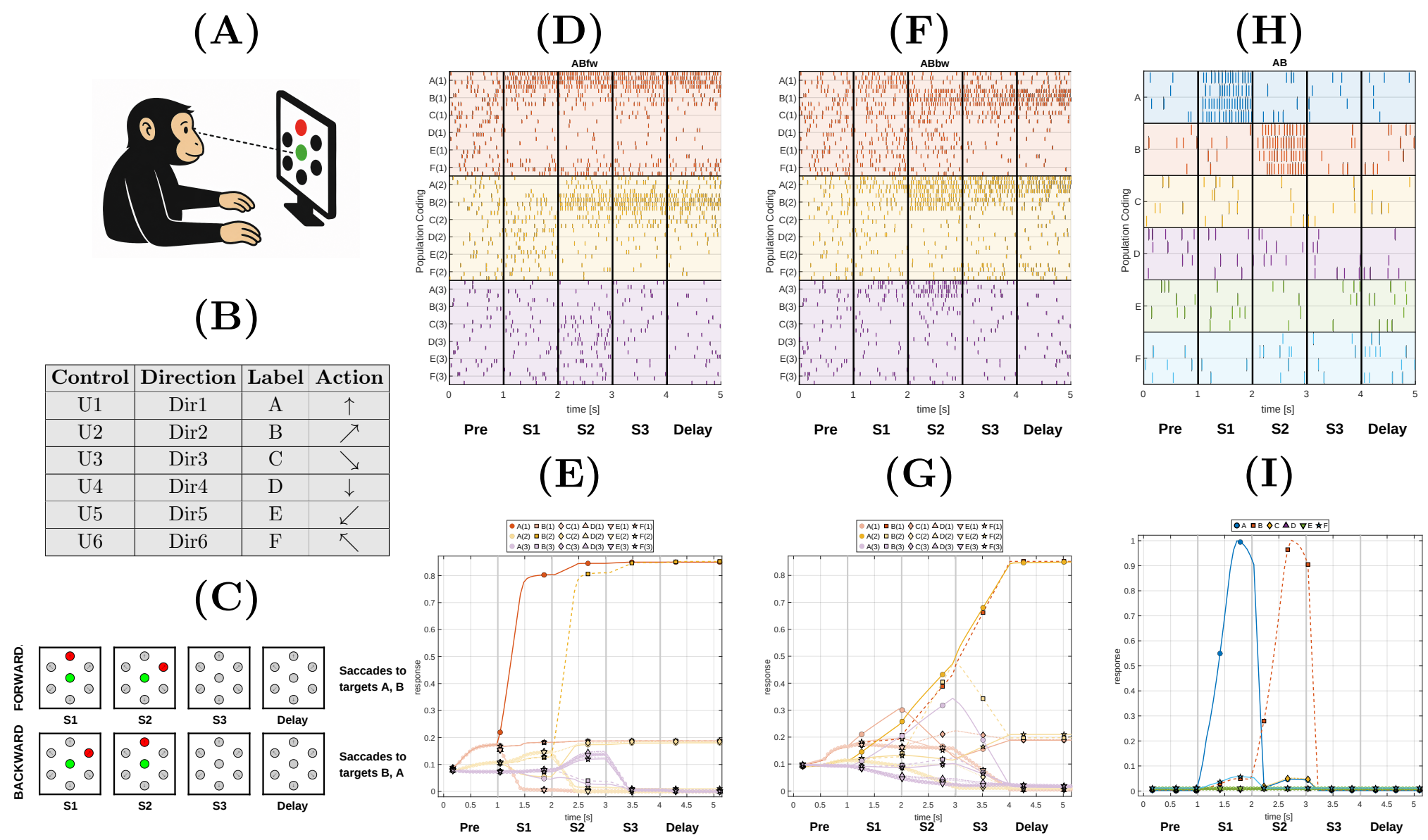

Control	Direction	Label	Action
U1	Dir1	A	↑
U2	Dir2	B	↗
U3	Dir3	C	↘
U4	Dir4	D	↓
U5	Dir5	E	↙
U6	Dir6	F	↖

Figure S3 Extended Simulation (3): inferring forward and backward sequences of two targets. This figure refers to the variable-length saccade experiment of (Chen et al., 2024), in which monkeys infer and maintain a sequence of saccades to be executed later in either forward or backward order. Here, the sequence length is two: targets are presented at S1 and S2, S3 is omitted, and the inferred sequence is maintained during the delay period. This simulation complements the main-text examples, where the sequence length is three. Panels (A) and (B) show the saccade task and the mapping between control states, target labels, and movement directions. Panel (C) illustrates the two-target trial structure. Panels (D) and (E) show a Forward-context simulation: (D) is a raster plot of simulated neural activity, with four neurons coding each of the six control states (targets A–F) at Level 2 and at each of the three sequence positions; (E) shows the same activity plotted as simulated firing rates. Panels (F) and (G) show the corresponding Backward-context simulation in the same format. Panels (H) and (I) show Level 1 activations during sequence observation, again in raster and firing-rate format. In this variable-length task, the execution context is known from the beginning of the trial; the policy prior therefore selects the relevant forward or backward policy subset at the outset while maintaining equal prior mass over sequence lengths 1, 2, and 3.

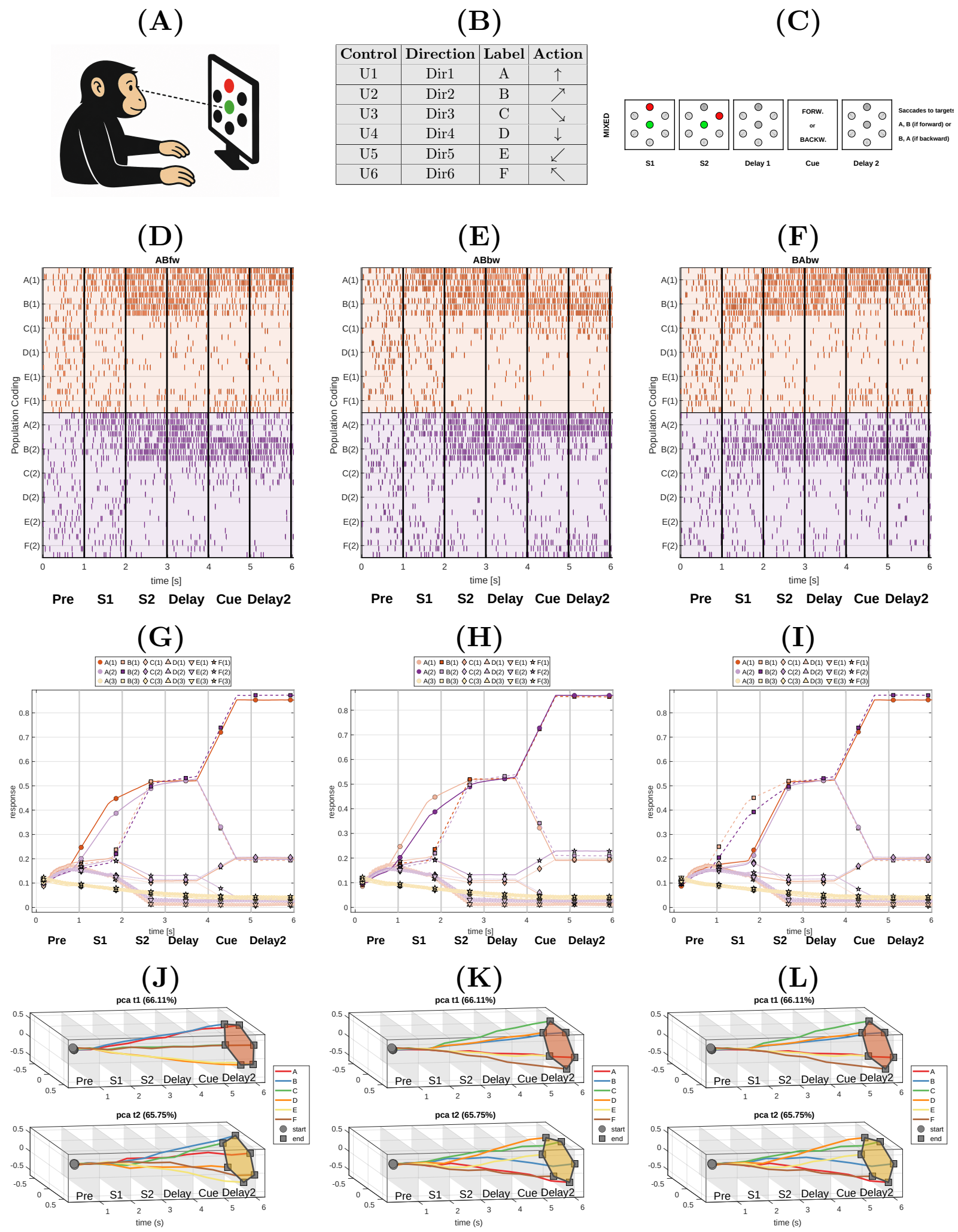

Figure S4 Extended Simulation (4): equal prior over *Forward* and *Backward* policies in the fixed-length saccade task. This figure follows the same task structure as the fixed-length saccade experiment of (Chen et al., 2024), in which monkeys infer and maintain a sequence of two targets and later execute it either forward or backward depending on a cue observed after the targets. In the main simulation, *Forward* policies are assigned a strong prior advantage before the cue; here, *Forward* and *Backward* policies are instead given equal prior weight. Panels (A)–(C) summarize the task structure corresponding to the explanatory panels of Figure 5 in the main text: two targets are presented sequentially at S1 and S2, followed by a delay period and an instruction cue. Panels (D)–(F) show raster plots for three example cases: AB in the forward condition, AB in the backward condition, and BA in the backward condition. Panels (G)–(I) show the corresponding simulated firing rates. Panels (J)–(L) show the corresponding trajectories of the first two principal components. The equal-prior variant produces a more symmetric inferential behaviour across forward and backward conditions than the empirically motivated main-text simulation. The coexistence of two different targets within the same memory subspace does not imply a decoding failure in this framework, because the plan is inferred rather than read out by a fixed decoder; the coexistence of alternatives reflects inference under equal prior probability, and the correct plan is recovered once the forward or backward cue is observed.

Supplemental references

Chen, J., Zhang, C., Hu, P., Min, B., & Wang, L. (2024). Flexible control of sequence working memory in the macaque frontal cortex. *Neuron*, 112(20), 3502–3514.

Mushiake, H., Saito, N., Sakamoto, K., Itoyama, Y., & Tanji, J. (2006). Activity in the lateral prefrontal cortex reflects multiple steps of future events in action plans. *Neuron*, 50(4), 631–641.

Parr, T., Pezzulo, G., & Friston, K. J. (2022). *Active inference: the free energy principle in mind, brain, and behavior*. MIT Press.

Xie, Y., Hu, P., Li, J., Chen, J., Song, W., Wang, X.-J., Yang, T., Dehaene, S., Tang, S., Min, B., et al. (2022). Geometry of sequence working memory in macaque prefrontal cortex. *Science*, 375(6581), 632–639.
